# Crumbs, Moesin and Yurt regulate junctional stability and dynamics for a proper morphogenesis of the *Drosophila* pupal wing epithelium

**DOI:** 10.1038/s41598-017-15272-1

**Published:** 2017-12-01

**Authors:** Pauline Salis, Francois Payre, Philippe Valenti, Elsa Bazellieres, André Le Bivic, Giovanna Mottola

**Affiliations:** 1 Aix-Marseille Université, CNRS UMR 7288, Developmental Biology Institute of Marseille Luminy (IBDM), Marseille, France; 2 Centre de Biologie du Développement, CNRS UMR5547, Université Paul Sabatier, Toulouse, France; 3 Present Address: UMR CNRS 7232 OOB, Université Pierre et Marie Curie, Banyuls-sur-Mer, France; 4 Laboratory of Biochemistry, La Timone University Hospital, Marseille, France; 5 Aix-Marseille University, UMR MD2 and IRBA, Marseille, France

## Abstract

The Crumbs (Crb) complex is a key epithelial determinant. To understand its role in morphogenesis, we examined its function in the *Drosophila* pupal wing, an epithelium undergoing hexagonal packing and formation of planar-oriented hairs. Crb distribution is dynamic, being stabilized to the subapical region just before hair formation. Lack of *crb* or *stardust*, but not *DPatj*, affects hexagonal packing and delays hair formation, without impairing epithelial polarities but with increased fluctuations in cell junctions and perimeter length, fragmentation of adherens junctions and the actomyosin cytoskeleton. Crb interacts with Moesin and Yurt, FERM proteins regulating the actomyosin network. We found that Moesin and Yurt distribution at the subapical region depends on Crb. In contrast to previous reports, *yurt*, but not *moesin*, mutants phenocopy *crb* junctional defects. Moreover, while unaffected in *crb* mutants, cell perimeter increases in *yurt* mutant cells and decreases in the absence of *moesin* function. Our data suggest that Crb coordinates proper hexagonal packing and hair formation, by modulating junction integrity via Yurt and stabilizing cell perimeter via both Yurt and Moesin. The *Drosophila* pupal wing thus appears as a useful system to investigate the functional diversification of the Crb complex during morphogenesis, independently of its role in polarity.

## Introduction

**Figure 1:**
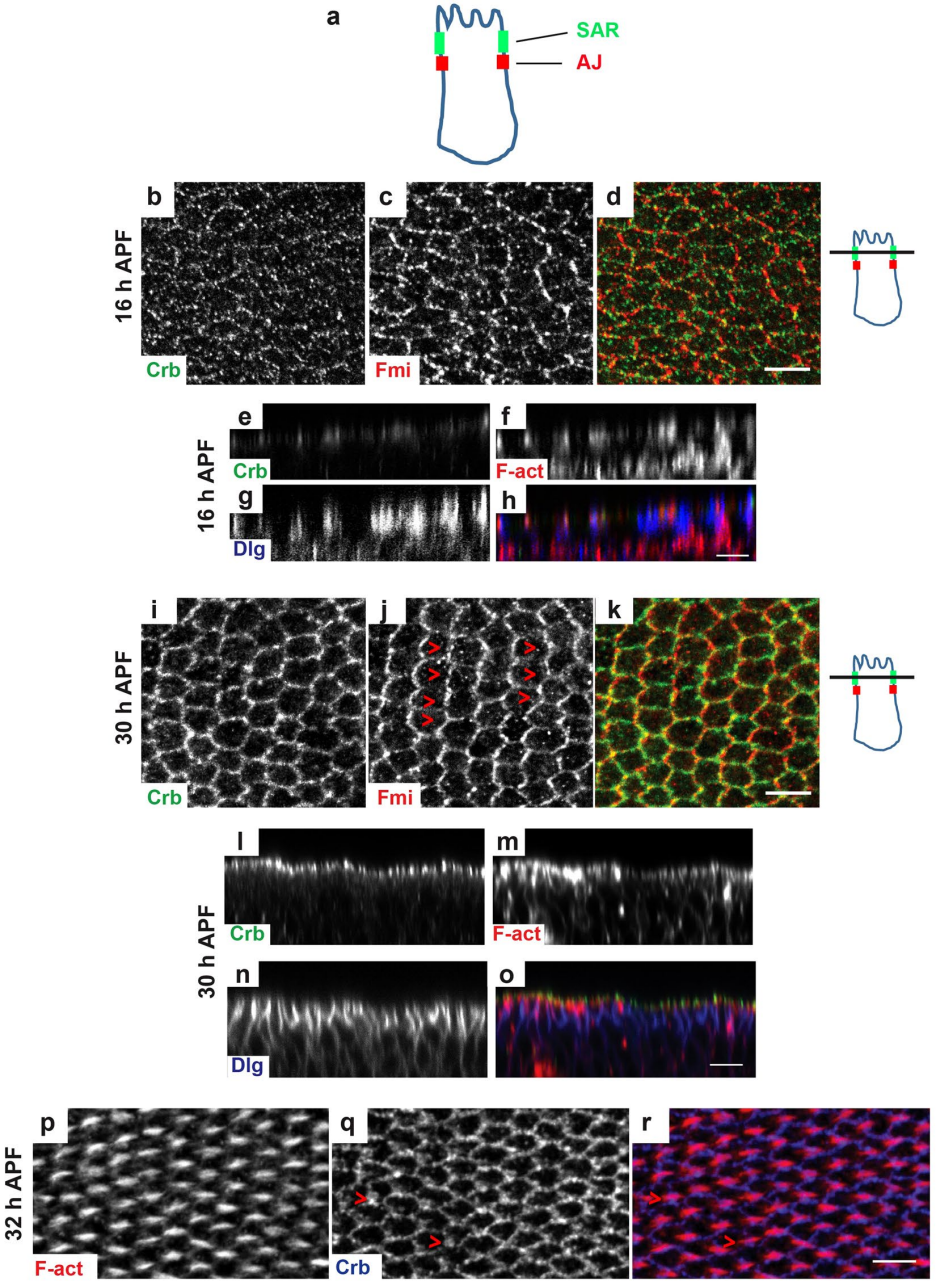
Crb displays a dynamic redistribution during pupal wing development. (**a**) Schematic drawing of a *Drosophila* epithelial cell, showing the position of the subapical region (SAR, in green) and of the adherens junctions (AJ, in red). (**b**–**d** and **i**,**k**) Crb (green) and Fmi (red) distribution in pupal wings at 25 °C at 16 h (**b**–**d**) or 30 h (**i**,**k**) APF; Red arrowheads in panel J show the Fmi zig-zag pattern oriented orthogonally to the PD axis. (**e**–**h** and **l**–**o**) Orthogonal sections of pupal wings at 16 h (**e**–**h**) or 30 h APF (**l**–**o**) stained for Crb (green), F-actin (red) and Dlg (blue). (**p**–**r**) Pupal wing at 32–34 h APF stained for Crb (blue) and F-actin (red). Red arrowheads in panel Q show Crb accumulation at the bottom of emerging hair. On the right of panels B–D and I–K drawn orthogonal views of a wing epithelial cell where the focal plane positions of the confocal image projections in the left panels are indicated (black line). All images are maximal projections of 2 up to 6 optical sections (every 0.2 μm). Distal is right, proximal left. Scale bar: 10 μm.

The type I transmembrane protein Crumbs (Crb) is a key regulator of epithelial cell integrity, which has been strongly conserved across evolution^1^. In most fly epithelia, Crb localizes to a subapical region (SAR), a membrane region positioned just above adherens junctions (AJs) [refs^2–4^ and Fig. 1a], where it forms a complex with the intracellular adaptor Stardust [Sdt] (Pals1 in Vertebrates) and DPatj^5,6^. Crb has been initially identified in flies for its role in maintaining epithelial organization^7^ and then in the expansion of the apical membrane upon overexpression^8^. These results demonstrate the key role of Crb in the organization of the apical domain, as further supported by studies in vertebrates [reviewed in refs^9–11^]. During later *Drosophila* development, Crb is involved in the positioning and stability of adherens junctions^12,13^. Crb is also connected to the actin cytoskeleton by its intracellular FERM-binding domain that interacts with three actin-binding proteins: Moesin (moe)^14^, β_H_-spectrin^14^ and Yurt^15^. Moe and Yurt negatively regulate Crb association to the membrane in some epithelia^15,16^. Recent evidence shows that Crb regulates actomyosin dynamics specifically via Moe, during dorsal closure in the embryo^17^ and for the morphogenesis of the adult follicular epithelium^16^. Therefore, Crb sits at a key position at physical/functional intersection of the apical membrane domain, adherens junctions and actin cytoskeleton. Because *crb* mutant embryos usually present strong apical-basal (AP/BL) polarity defects, whether and how Crb could regulate apical organization during morphogenesis yet remains poorly understood.

The *Drosophila* pupal wing represents a useful model to address the role of Crb in epithelia morphogenesis. Crb is not essential for AP/BL polarity in the third instar imaginal disc, the larval epithelium that develops into the pupal wing^18,19^. In the absence of intense cell proliferation, the pupal wing epithelium undergoes dramatic cell rearrangements, leading to a characteristic hexagonal cell packing. Hexagonal packing requires reorganization of the actin cytoskeleton and AJs, as well as polarized localization of proteins involved in Planar Cell Polarity (PCP)^20–22^. This eventually results in a monolayered epithelium, differentiating a single F-actin-rich prehair (trichome) at the distal vertex of each cell, with a defined proximal-distal (P/D) orientation. Mutations in genes that control wing morphogenesis lead to hair defects, as easily seen in the adult^23–25^. For instance, the loss-of-function of key cytoskeleton regulators such as Zipper (Myosin II heavy chain) leads to cells forming multiple hairs^26–32^. Thus, the apico-basal polarity, junction organization and apical cytoskeleton remodeling are intimately interconnected during wing differentiation^33,34^.

In this study, we investigated the role of Crb, Sdt and DPatj during pupal wing development. We found that both Crb and Sdt (but not DPatj) play a role in epithelial morphogenesis that is independent of the apico-basal or PCP pathways. Our data further indicate that Crb is necessary for the integrity and stability of E-cadherin (E-cad) and actomyosin at the adherens junctions at the end of hexagonal packing, a function likely mediated by Yurt. In addition, our results suggest a role of Crb in modulating opposed Moesin- and Yurt-dependent mechanisms for the regulation of the cell perimeter.

## Results

### Crb redistributes to the subapical region during pupal wing development

Although the putative function of Crb has never been examined in the development of adult wings that occurs during pupal stages, previous studies have noticed that Crb accumulates at the SAR of epithelial cells in the larval wing imaginal discs^35–37^, suggesting that Crb regulates epithelium morphogenesis at later stages of development.

As a first step, we investigated whether and where Crb accumulates in developing pupal wing cells. Pupal wing development comprises three major morphogenetic events: 1) cell packing [10–28 h after puparium formation (APF)], resulting from E-cad-dependent remodeling of cell contacts that allows irregularly shaped cells turning into honeycomb-packed hexagons; 2) establishment of the P/D axis (28–30 h APF), as visualized by typical pattern of the PCP protein Flamingo (Fmi) at the SAR; 3) a spectacular rearrangement of the actin cytoskeleton (32–34 h APF) for the formation of prehairs [reviewed in^22,38,39^].

We found that Crb is expressed throughout all morphogenetic stages at the apical region of pupal wing cells and displays highly dynamic redistribution during their differentiation (Fig. 1). During hexagonal packing (at 16 h APF), Crb exhibits a punctuated pattern both intracellularly and at the SAR and localizes just above a basolateral membrane marker, Discs large 1 tumor suppressor (Dlg)^40^ (Fig. 1b–h). Then, when the P/D axis is established, Crb distribution is less punctuated and more associated to the SAR (Fig. 1i–o), as in wing imaginal discs^35–37^. While Fmi becomes restricted to the P/D boundaries of the SAR (Fig. 1j and k, arrowheads), Crb has a uniform redistribution along the membrane (Fig. 1i and k). We found that Crb localization at the SAR is dependent of Sdt, since the genetic nullification of Sdt leads to the loss of Crb staining (Fig. S1a–c), as also reported in other epithelia^41^. In contrast, whereas the absence of DPatj leads to a decrease in Crb levels, it is not sufficient to prevent Crb accumulation at the SAR (Fig. S1g–i). Finally, when prehair formation takes place, Crb continues to associate to SAR and occasionally accumulates at the distal vertex of the cell (Fig. 1p–r, arrowheads).

Hence, Crb is expressed and apically localized within developing pupal wing cells, with a specific redistribution to the SAR coinciding with the end of hexagonal packing. These data therefore suggested that the Crb complex contributes to wing cell morphogenesis, a hypothesis we next evaluated using genetic analysis.

### Crb is not required for apical/basal and planar polarities of the pupal wing epithelium, but participates in prehair formation and hexagonal packing during morphogenesis

**Figure 2:**
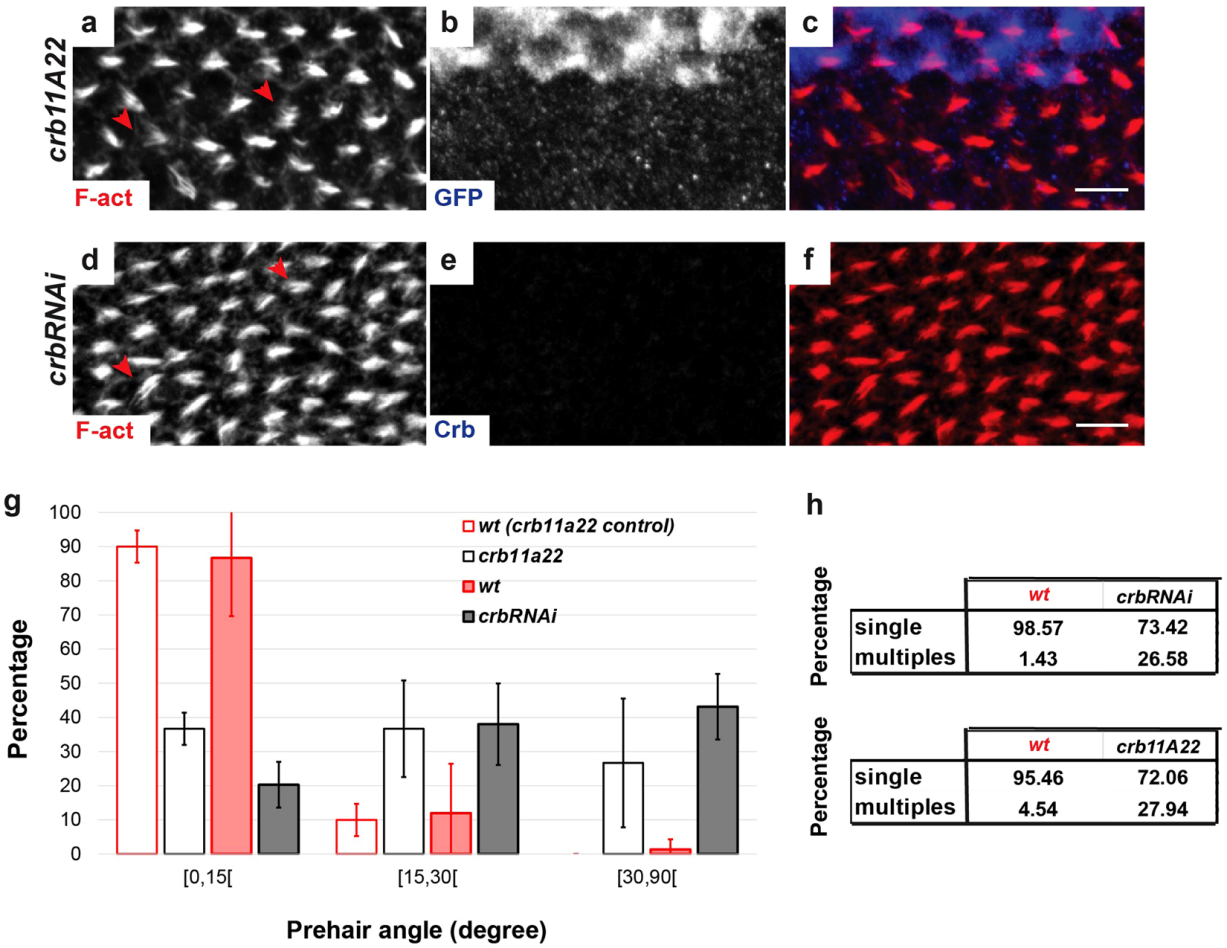
Depletion of *crb* expression affects prehair morphogenesis. (**a**–**f**) *crb* ^*11A22*^ clones (**a**–**c**) and *crbRNAi* cells (**d**–**f**) in pupal wings at 32–34 h APF, indicated by the absence of GFP or Crb (blue), respectively, stained for F-actin (red). Red arrowheads in panels A and D show double or triple mis-oriented prehair in *crb* mutant cells. Scale bar: 10 μm. (**g**) Histogram of angles (degrees) between prehair and the L3 vein in *wt*, *crbRNAi, crb* ^*11A22*^ cells at 32–34 h APF. (**h**) Tables showing the percentage of single and multiples prehair in *wt*, *crbRNAi, crb* ^*11A22*^ cells at 32–34 h APF.

To investigate the putative role of Crb in pupal wing morphogenesis, we examined prehair formation in tissues manipulated to inactivate the individual function of *crb, sdt* and *dPatj* using targeted expression of RNAi, as well as mosaic clones of null alleles (Figs 2 and S1). Compared to neighboring *wild type* (*wt)* cells (Figs 1p and 2a), cells homozygous for *crb* ^*11A22*^ (marked by loss of *GFP*, Fig. 2a–c) or expressing *crb*RNAi (Fig. 2d–f), exhibit a disorganized pattern of prehairs. Indeed, while ~90% of prehairs in *wt* tissue points to the P/D axis (with an angle between the prehair and the P/D axis of 0 to 15 degrees), prehairs alignment presents a higher variability in *crb* mutants cells, with an angle ranging from 0 to 90 degrees (Fig. 2g). Moreover, multiple prehairs per cell are often observed in *crb* mutant cells (Fig. 2a and d, arrowheads). We quantified 26.58% and 27.94% of cells showing multiple hairs in *crbRNAi* and *crb* ^*11A22*^ cells, respectively, compared to controls where multiple prehair cells are lower than 5% [n_wings_ = 5, n_cells_ = 100] (Fig. 2h). These defects therefore reinforce the hypothesis that Crb is required for the proper morphogenesis of pupal wing cells.

Consistently, wing cells homozygous for a null allele of *sdt* (*sdt* ^*k85*^) show a similar phenotype to that observed in the absence of Crb (Fig. S1d–f). In contrast, no defects in prehair formation are observed in *DPatj* ^*53*^ mutant cells (Fig. S1j–l). Surprisingly, wings containing *crb* ^*11A22*^ clones or *crbRNAi* knockdown produce normal-looking adult hairs (data not shown). It has been proposed that hair morphogenesis starts with the formation of multiple bundles of actin near the distal vertex, which merge over time to form a single hair^20,26^. The formation of multiple prehairs in *crb* mutants that eventually resolve into adult hairs of normal morphology, suggested a delay rather than an impairment of adult hair formation upon Crb depletion. Remarkably, mutations in *mwh*, a negative regulator of the actin cytoskeleton, induce a similar delay in hair development^42^. Therefore, our results argue for a cell-autonomous role of Crb in the regulation of hair development.

Crb is essential for apical domain organization and AP/BL polarity in most epithelia analyzed^8,43^. Therefore, we addressed whether prehair defects were associated with an alteration of cell polarity. Analysis of the basolateral membrane marker Dlg^40^ in *crbRNAi* and *crb* ^*11A22*^ cells does not show any changes compared to *wt* tissues (Fig. S2a–l and data not shown). Orthogonal sections show that the localization of AJs, visualized by E-cad staining, is indistinguishable in *crbRNAi* cells from *wt* cells (Fig. S2g–l). Also, no differences in cell height are detected between *wt* and *crbRNAi* cells (Fig. S2g–l). These observations argue that AP/BL polarity in *crb* mutants is not affected, as opposed to other *Drosophila* tissues^8,43^.

Although a function of Crb in PCP has not been previously addressed, the *crb* mutant phenotype in prehair formation could be linked to defects in the establishment and/or maintenance of the P/D axis, a hypothesis we next assayed through examining the distribution of Fmi. As in *wt* cells, the asymmetric localization of Fmi at the proximal-distal cell boundaries (giving rise to a characteristic zig-zag pattern of Fmi) is unchanged (red dots) in *crbRNAi* cells (Fig. S2m–r, red dots) and in *crb* ^*11A22*^ clones (not shown), showing that PCP polarity is not disrupted upon *crb* depletion.

Taken together, our data therefore indicate that Crb is not involved in the maintenance of AP/BL polarity or the establishment of PCP in pupal wing cells, but Crb is instead required for proper prehair formation through an independent pathway.

**Figure 3:**
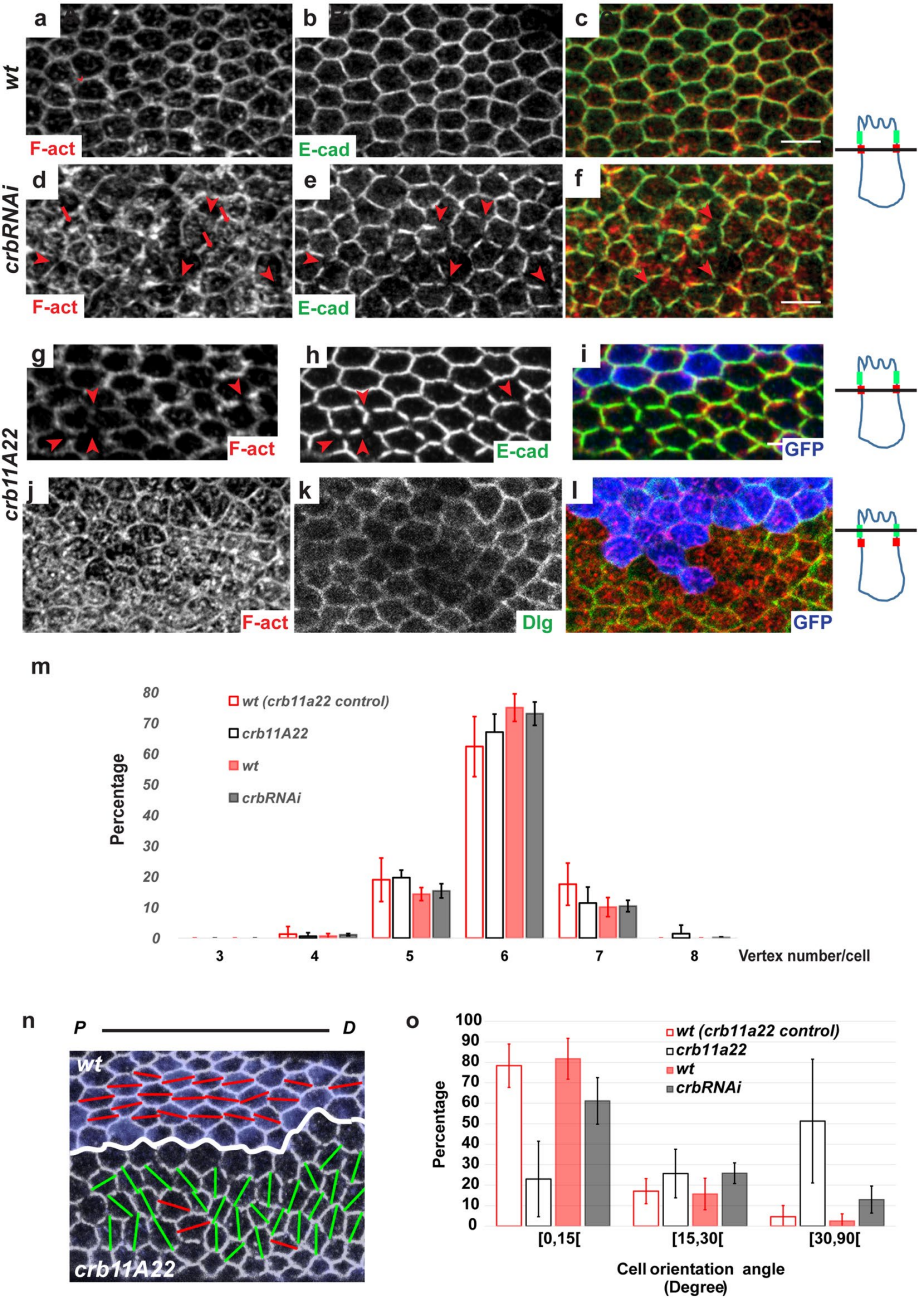
Crb is required for the integrity of adherens junctions and of the F-actin cytoskeleton belt and for the P/D orientation of epithelial cells during hexagonal packing (**a–l**) *wt* (**a–c**), *crbRNAi* (**d–f**) and *crb* ^*11A22*^ cells (**g–l**) in pupal wings at 28–30 h APF, stained for F-actin (red) and E-cad (green, **a–i**) or Dlg (green, **j–l**). *crb* ^*11A22*^ clones are indicated by the absence of GFP (blue). Red arrowheads in d, e, g and h show cortical gaps devoid of F-actin and E-cad. Red arrows in d show the intracellular accumulation of F-actin. On the right of panels a-l, drawn orthogonal views of a wing epithelial cell where the focal plane positions of the confocal image projections in the left panels are indicated (black line). All images are maximal projections of 2 up to 6 optical sections (every 0.2 mm). Distal is right, proximal left. (**m**) Histogram of vertex number in wt, *crbRNAi*, *crb* ^*11A22*^ cells at 28–30 h APF. Note that the percentage of cells with 6 vertices (hexagons) is similar between wt and *crb* mutant tissues. Bars indicate mean values ± SEM. n_wings_ =9, n_cells_ =180 *crbRNAi* and *wt* *crbRNAi* were analyzed. (**n**) *wt* (on the top) and *crb* ^*11A22*^ (on the bottom) cells at 30–34 h APF marked with E-cad. Cell orientation angle corresponding to the absolute angle between the longest axis of the cell and the vein L3 was drawn with bars in each cell. Red bars correspond to an angle <25° whereas green bars correspond to an angle >25°. (**o**) Histogram of the distribution of cell orientation angle in *crbRNAi* and its *wt* control, *crb* ^*11A22*^ and its *wt* twin clone cells at 30–34 h APF. Note that the percentage of cells with an orientation angle under 15° is lower in *crb* mutants than in *wt* cells. Bars indicate mean values of cells percentage ± SEM and statistical significance was analyzed by Student’s t-test {*crb* ^*11A22*^[0,15[=23.0% ± 10.65, versus *wt*[0,15[=78.6% ± 6.11; *crb* ^*11A22*^[15,30[=25.7% ± 6.80, versus *wt*[15,30[=17.1% ± 3.55; *crb* ^*11A22*^[30,90[=51.3% ± 17.45, versus *wt*[30,90[=4.6% ± 3.17; n_wings_=3, n_cells_=60, P < 0.05; *crbRNAi*[0,15[=61.2% ± 3.80, versus *wt*[0,15[=80.9% ± 3.74; *crbRNAi*[15,30[=25.9% ± 1.68, versus *wt*[15,30[=18.9% ± 4.20, *crbRNAi*[30,90[=13.0% ± 2.19, versus *wt*[30,90[=2.8% ± 1.31; n_wings_=9, n_cells_=180; P < 0.05]. Scale bar: 10 mm.

The proper formation of prehairs in pupal wing cells requires hexagonal packing^22,38,39^. During hexagonal packing cells change their shape and, concomitantly, increasingly point to the P/D axis in response to tissue stretching^44,45^. At the end of tissue remodeling (28–30 h APF), all cells are turned into a hexagon, displaying regular vertex to vertex distances and highly similar cell perimeters (Fig. 3a–c). This results in an ordered honeycomb-like pattern of cell junctions, the asymmetric distribution of PCP components along the P/D cell sides defining the distal-most apical vertex where prehairs start growing and become aligned on each other.

We observed that the inactivation of *crb* strongly disrupts tissue rearrangement, with a loss of the honeycomb-like pattern (Figs 3e and S2p). Quantification of the apical cell perimeter does not detect significant differences between *wt* and *crb* mutant cells (Fig. S3a, see Figure legend), and most *crb* mutant cells retain six vertices (Fig. 3m), without significant modification of the average vertex to vertex distance (Fig. S3b, see Figure legend). Next, we addressed whether hexagons point to the P/D axis by measuring the orientation of hexagonal cells, which we defined by the angle between the longest axis of a fitted ellipse and the vein 3 that is parallel to the P/D axis (See Materials and methods and Fig. 3n). Remarkably, we found that the alignment of hexagonal cells was altered in *crb* mutants. Indeed, while >80% of control cells displays an average angle ≤15 degrees, *crb* mutant cells exhibit a higher variability in their orientation and pointing to the P/D axis (Fig. 3n). These results thus suggest a role of Crb in regulating hexagonal packing through the P/D alignment of cells.

### Crb is required during wing morphogenesis for the integrity and stability of adherens junctions and circumferential actomyosin belt

**Figure 4:**
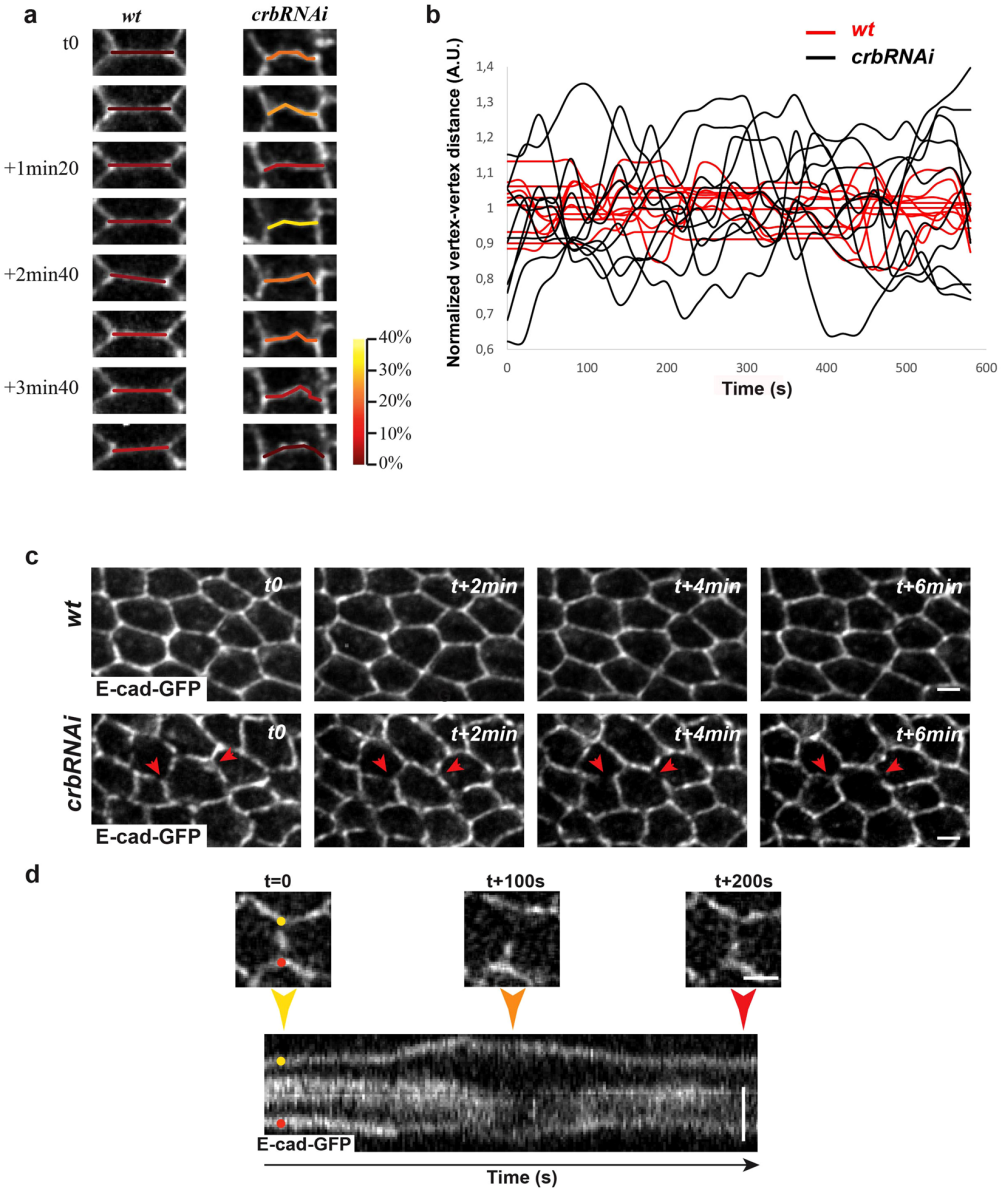
Crb is required for the stability of vertex-vertex length fluctuations. (**a**–**d**) *wt* and *crbRNAi* pupal wing cells expressing E-cad::GFP at 28–30 h APF were imaged *in vivo* to follow the evolution of vertex-vertex length (**a**,**b**) and the evolution of gaps devoid of E-cad (**c**,**d**). (**a**) Example of variation of vertex-vertex distance length. Vertex-vertex distance is color-coded (heat map from red to yellow) based on the percentage of vertex-vertex distance decrease calculated with respect to the vertex-vertex distance captured during imaging. (**b**) Graph of the evolution of vertex-vertex length variation amplitude expressed in A.U. in 10 *wt* (red) and 10 *crbRNAi* (black) cells. Each distance is normalized by its average length over time and expressed in arbitrary units (A.U.). (**c**) Example of the evolution of gaps devoid of E-cad (red arrowheads) in *wt* and *crbRNAi* pupal wing cells. (**d**) Higher magnification of a cell-cell contact imaged over the indicated time and its associated kymograph. Scale bar: 0.5 μm.

The defects in prehair orientation and hexagonal alignment of Crb mutant cells are also associated to strong alterations of cells junctions and actin cytoskeleton. Cell junctions are often wiggly (Fig. 4a) and show higher length fluctuations amplitude over time when compared to *wt* cells [Amplitude of junction length fluctuation over time in *wt* cells = 0.10 ± 0.01 A.U. versus *crbRNAi* = 0.17 ± 0.01 A.U. (n_wings_ = 5, n_cells_ = 40), P < 0.0001] (Fig. 4b), suggesting altered cortical tension. Consistently, the apical surfaces of *crb* mutant cells constrict and expand with higher amplitude than in *wt* cells [mean of cell perimeter length fluctuation over time in *wt* cells = 0.30 ± 0.02 A.U. versus *crbRNAi* = 0.78 ± 0.07 A.U., n_wings_ = 5, n_cells_ = 10, P < 0.0001] (Fig. S3c and d). We also observed a fragmentation of E-cad staining in both *crbRNAi* (Figs 3e and 4c and d, red arrowheads and movie 1) and *crb* ^*11A22*^ cells (Fig. 3g–i, red arrowheads). Live imaging of *crbRNAi* cells revealed that these gaps are transient (Fig. 4c and d, arrowheads, and movie 1), quickly forming when junctions expand and disappearing when junctions retract.

The defects observed in *crb* mutant cells supported an alteration in membrane tension, which results from the interaction between the E-cadherin adhesion system and the contractile actomyosin cytoskeleton [reviewed in ref.^46^]. The existence of E-cad and actomyosin clusters involved respectively in junctional stability and contractility has previously been described in mammalian cells^47,48^. Consistently, we observed that the junctional F-actin was abnormally fragmented in *crb* mutant cells, and regions lacking junctional F-actin are the ones also devoid of E-cad (Fig. 3d–j, arrowheads). Further aberration of the circumferential actomyosin belt was also visualized at the SAR, where F-actin is delocalized intracellularly in the subapical cytoplasm (Fig. 3d, arrows, and Fig. S2q). We did not, however, detect obvious alteration of basolateral F-actin (Fig. S2b and e), consistent with a specific role of Crb at the SAR. Similar actin defects are also observed in *crb* ^*11A22*^ null clones (Fig. 3j–l).

Taken together, these data show that the absence of Crb causes a correlated fragmentation of the cortical actin cytoskeleton and AJs, and a larger fluctuation in both junction length and apical cell perimeter. These results thus suggest that Crb stabilizes E-cad and the actin cytoskeleton at the adherens junctions to regulate proper hexagonal packing.

### Loss of the FERM protein Moesin alters cell perimeter and F-actin accumulation but not the circumferential actomyosin belt and adherens junctions

**Figure 5:**
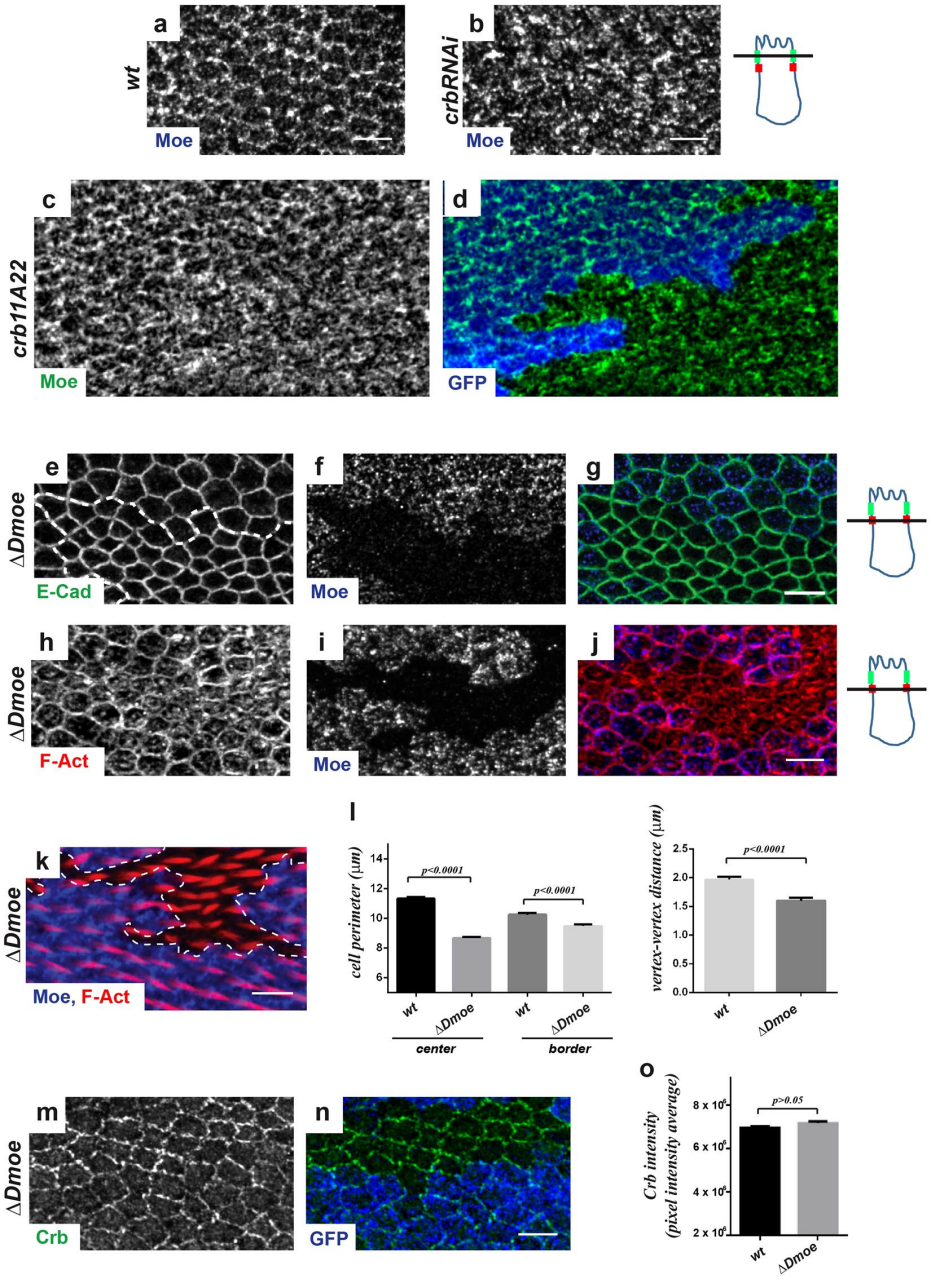
Loss of *Moesin* induces apical cell perimeter constriction, but does not affect adherens junctions. (**a**–**d**) Staining of Moe in *wt* (**a**), *crbRNAi* (**b**) or in *crb* ^*11A22*^ (**c**,**d**) pupal wing cells at 28–30 h APF. In *crb* ^*11a22*^ the *wt* twin clone is labelled by GFP expression (blue), Moe staining is in green (**c**,**d**). (**e**–**k**) Pupal wings containing *Δmoe* clones at 28–30 h (**e**–**j**) or 32–34 h (**k**) APF stained for E-cad (green), F-actin (red) or Moe (blue). E-cad and F-actin staining revealed defects in cell perimeter (**e**,**h**) or apical F-actin redistribution (**h**). On the right of panels a-j, drawn orthogonal views of a wing epithelial cell where the focal plane positions of the confocal image projections in the left panels are indicated (black line). All images are maximal projections of 2 up to 6 optical sections (every 0.2 μm). Distal is right, proximal left. Scale bar: 10 μm. (**l**) Quantification of cell perimeter length (Left) and vertex-vertex distances (Right) (μm) in *wt* and *Δmoe* clones (in the center of the clone and along the border of the clone) in wings at 28–30 h APF. Bars indicate mean values ± SEM and statistical significance was analyzed by Student’s t-test [*Δmoe* clones versus *wt* cells, n_cells_ = 200, P < 0.0001, see the main text]. (**m**,**n**) Pupal wings containing *Δmoe* clones at 28–30 h APF stained for Crb (green). Moe depletion is revealed by the absence of GFP (blue). Distal is right, proximal left. Scale bar: 10 μm. (**o**) Quantification of Crb staining at the SAR in *wt* and *Δmoe* clones (in the center of the clone and along the border of the clone) in pupal wings at 28–30 h APF. Crb intensity per length unit (pixel intensity average) at the apico-lateral cortex was calculated and expressed in A.U. Bars indicate mean values of intensity ± SEM and statistical significance was analyzed by Student’s t-test [*Δmoe* = 7.18 ± 0.08 × 10^6^ A.U., versus *wt* = 6.96 ± 0.08 × 10^6^ A.U., n_wings_ = 5, n_cells_ = 70; P > 0.05].

In order to elucidate how Crb regulates the circumferential actomyosin belt and adherens junctions, we analyzed the contribution of Moe, a FERM protein that interacts with Crb and regulates actin-based cell shape^14,49–51^. Interestingly, Moe mostly localizes at the SAR at the end of hexagonal packing (Fig. 5a and c) and its localization is perturbed upon Crb depletion (Fig. 5b–d). To determine whether *moe* had a role in pupal wing morphogenesis, we first analyzed an available strong allele, *moe* ^*PL106*^, resulting from a P-element insertion^49,50^. None of the defects observed for *crb* mutant cells were detected in clones of *moe* ^*PL106*^ cells. However, immuno-staining revealed remnants of Moe levels in *moe* ^*PL106*^ cells (Fig. S4e), precluding a definitive conclusion. We therefore generated a true *moe* loss-of-function allele, *Δmoe*, by inducing a targeted genomic deletion that removes most *moe* coding sequences (Fig. S4g and see Methods). Although Moe is clearly absent in mutant clones (Fig. 5f and i), cells lacking *moe* do not display the clear mis-organization of prehairs observed for *crb* mutants (Fig. 5k). However, we detected a strong apical constriction phenotype in *Δmoe* cells (Fig. 5e,h and Movie 2). Quantification of the cell perimeter of *Δmoe* cells localized in the middle of mutant clones indicated a strong decrease, compared to *wt* cells of the corresponding twin clones (*Δmoe* = 8.70 μm ± 0.10 versus *wt* = 11.30 μm ± 0.10, n_cells_ = 100, P < 0.0001; Fig. 5l). Interestingly, the decrease in cell perimeter is less pronounced for *Δmoe* cells positioned along the clone border (*Δmoe* edge cells = 9.50 μm ± 0.10, versus *wt* edge cells = 10.30μm ± 0.10, n_cells_ = 100, P < 0.001). The reduction in cell perimeter is also associated to a decrease in the average junction length (*Δmoe* = 1.60 μm ± 0.05, versus *wt* = 2.00 μm ± 0.05, n_cells_ = 100, P < 0.001; Fig. 5l). Crb levels at the SAR appeared increased in *Δmoe* cells (Fig. 5m,n). Nonetheless, this increase in Crb accumulation can be explained by the associated decrease in the perimeter of *Δmoe* cells. Indeed, Crb intensity per length unit (pixel intensity average) remains mainly unchanged in *Δmoe* cells (*Δmoe* = 7.18 ± 0.08 × 10^6^ A.U., versus *wt* = 6.96 ± 0.08 × 10^6^ A.U., n_wings_ = 5, n_cells_ = 70; P > 0.05, Fig. 5o). Moreover, an accumulation at the membrane is also observed for other proteins, such as E-cad and Sdt (Fig. S5a–f), as well as Fmi, despite the absence of a significate increase in Fmi intensity per length unit (*Δmoe* = 6.29 ± 0.08 × 10^6^ A.U. versus *wt* = 6.06 ± 0.08 × 10^6^ A.U., n_wings_ = 5, n_cells_ = 100; P > 0.05, Fig. S5g).

Consistently with the role of Moe as a regulator of apical actin, F-actin accumulates at the apical cytoplasm in *Δmoe* cells, while some signal could still be detected at the AJs (Fig. 5h). However, the distribution of both E-cad (Fig. 5e) and F-actin (Fig. 5h) is not fragmented and we observed no gaps in the adherens junctions in *Δmoe* pupal wing cells, as opposed to the defects featuring observed for *crb* mutant cells (see Movie 2).

We thus concluded that Crb controls Moe distribution at the SAR, suggesting a functional connection between these proteins. Our data yet indicate that Moe is not directly involved in the stabilization of adherens junctions, but instead it mainly acts to regulate the apical cell perimeter during pupal wing morphogenesis.

### The FERM protein Yurt is essential for the proper organization of the circumferential actomyosin belt and adherens junctions

**Figure 6:**
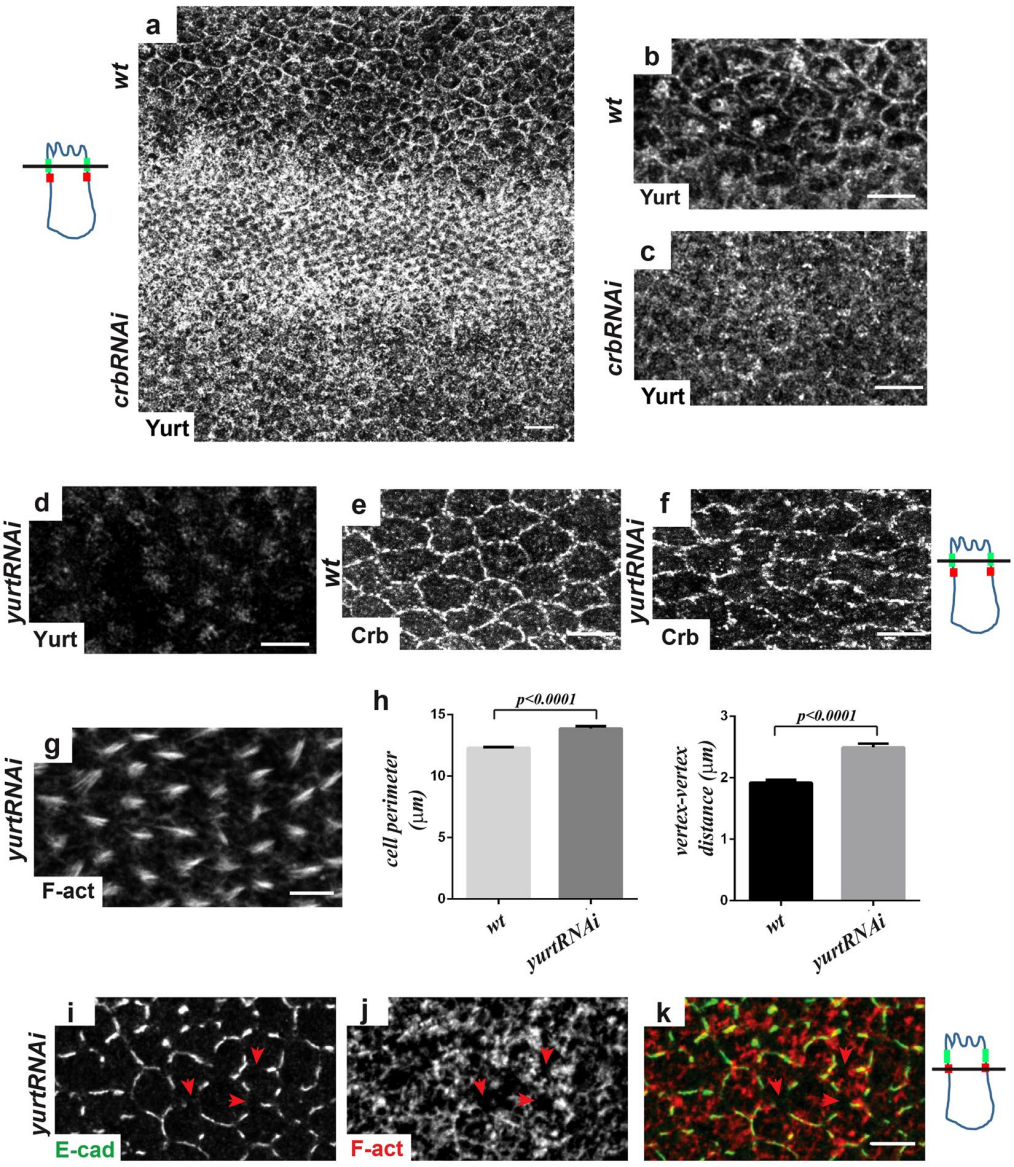
Loss of *yurt* induces cell perimeter expansion and affects the circumferential actomyosin belt and adherens junctions. (**a**–**d**) Staining of Yurt in *wt* (A, at the top of the wing), crbRNAi (**a**, at the bottom of the wing and **c**) and *yurtRNAi* (**d**) pupal wings (at 28–30 h APF). Higher magnifications of *wt* (**b**) and *crbRNAi* (**c**) tissues are shown. Note that while the cortical staining of Yurt disappears in *yurtRNAi* cells, the intracellular staining remained mainly unchanged suggesting that it is not specific (**d**). (**e**,**f**) Staining of Crb in *wt* (**e**) and *yurtRNAi* (**f**) pupal wings (28–30 h APF). Note that in *yurtRNAi* cells, although fragmented, Crb still associates to the SAR. (**g**) F-actin staining in *yurtRNAi* pupal wings at 32 h APF show defects in prehair organization. (**h**) Quantification of cell perimeter length and vertex–vertex distance (μm) in *wt* and *yurtRNAi* cells at 28–30 h APF. Bars indicate mean values ± SEM and significance was analyzed by Student’s t-test [*yurtRNAi* versus *wt* cells, n_cells_ = 200, P < 0.0001, see main text]. (**i**–**k**) Staining of E-cad (green) and F-actin (red) in *wt* and *yurtRNAi* pupal wings (28–30 h APF) reveals defects in junctional integrity in *yurtRNAi*. Red arrowheads show gaps devoid of F-actin and E-cad staining at the adherens junctions. On the left of panels A–C and on the right of panels d–f and i–k, drawn orthogonal views of a wing epithelial cell where the focal plane positions of the confocal image projections in the left panels are indicated (black line). All images are maximal projections of 2 up to 6 optical sections (every 0.2 μm). Distal is right, proximal left. Scale bar: 10 μm.

Crb directly binds via its cytoplasmic FERM-binding region to Yurt^52,53^, which is known to negatively control Crb association to tight junctions in mammals^15^. To decipher how E-cad and F-actin gaps at the AJs are produced independently of Moe, we focused on the possible involvement of Yurt in this process. Interestingly, we observed that Yurt associates to the SAR at the end of hexagonal packing (as revealed by Yurt staining and comparing *wt* and *yurtRNAi* cells, Fig. 6a,b and d). Yurt localization at the SAR is dependent on Crb because this staining is lost upon *crb* inactivation (Fig. 6b and c). In contrast to other tissues or developmental stages, *yurt* knockdown by two distinct RNAi does not affect Crb accumulation at the SAR, even if its distribution is slightly more discontinuous (Fig. 6e,f, and not shown). In contrast to the lack of Moe, Yurt depletion leads to a strong disorganization of prehairs (Fig. 6g) and to prominent junctional defects similar to those observed in *crb* mutants, *i.e*., a correlated disruption of E-cad and F-actin at adherens junctions that could explain the observed discontinuities for Crb distribution (Fig. 6i–k). A phenotype not seen in the absence of Crb, however, is an increase in the perimeter of *yurtRNAi* cells (13.90 μm ± 0.20 versus *wt* 12.30 μm ± 0.08; n_wings_ = 5, n_cells_ = 200, P < 0.001), as also supported by an increased mean distance between vertices (*yurtRNAi* = 2.50 μm ± 0.07 versus *wt* = 1.90 μm ± 0.05; n_wings_ = 5, n_cells_ = 200, P < 0.001, Fig. 6h).

**Figure 7:**
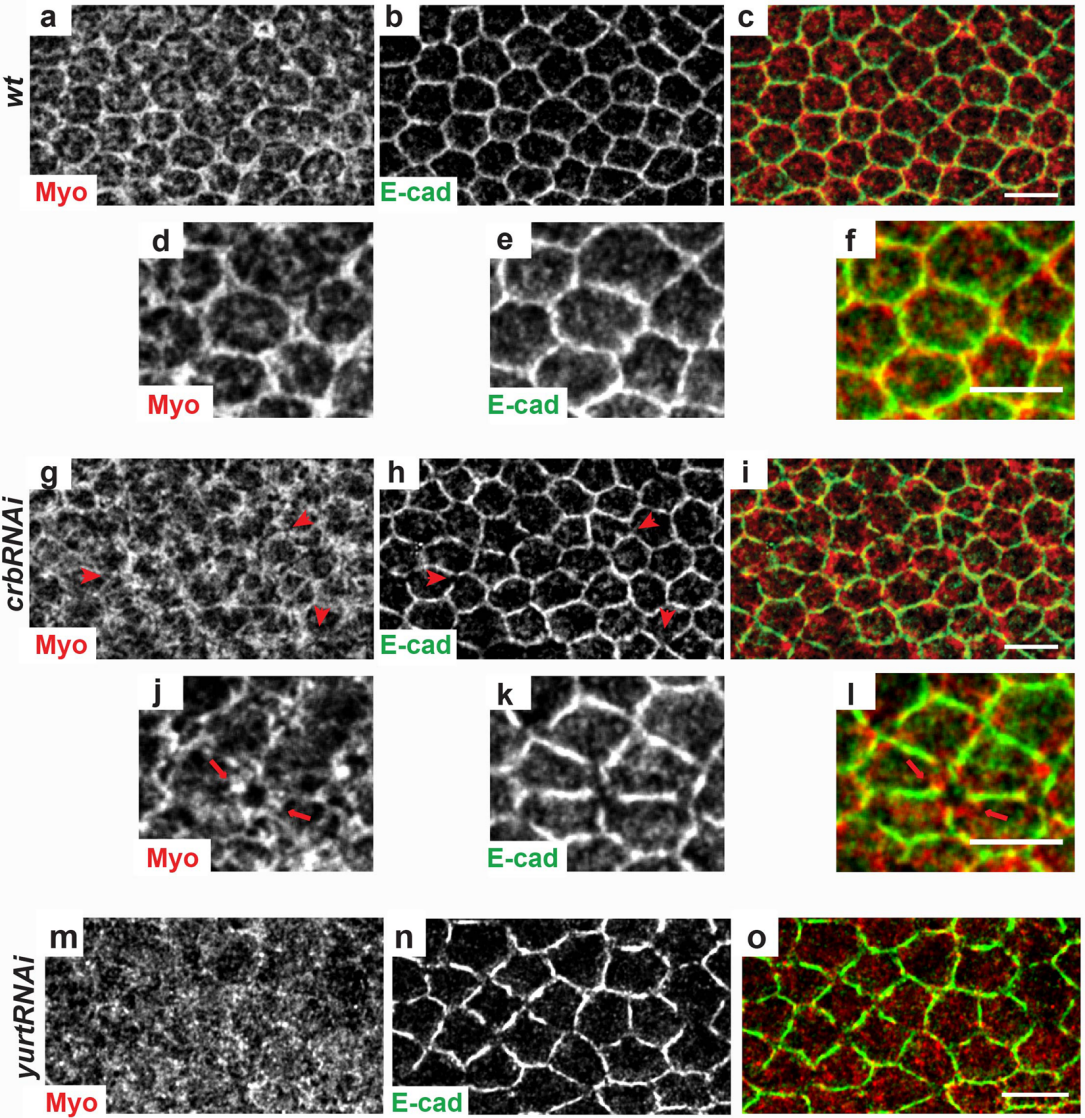
Myo2 distribution is affected in *crbRNAi* and *yurtRNAi* cells. *wt* (**a**–**f**), *crbRNAi* (**g**–**l**) and *yurtRNAi* (**m**–**o**) pupal wing cells at 28–30 h APF stained for E-cad (green) and Myo2 (red). Red arrowheads in g and h show gaps at the cortex devoid of E-cad staining. Red arrows in j and l show a gap at the cortex devoid of E-cad and surrounded by Myo2 accumulation. All images are maximal projections of 2 up to 6 optical sections (every 0.2 μm). Distal is right, proximal left. Scale bar: 10 μm.

Myosin II (Myo) has been implicated in the regulation of tension during epithelial morphogenesis and Myosin apical accumulation preceded the constriction and intercalation of embryonic cells^48,54,55^. Inactivation of *myosin II* (*zipper*) in wing cells leads to multiple hairs^26–30,32^. To better understand the cytoskeletal modifications observed in *crb* and *yurt* mutants, we next examined Myo distribution in these contexts (Fig. 7). In *wt* wing cells at the end of hexagonal packing Myo tightly associates to AJs and vertices (Fig. 7a–f), as previously shown in embryos^54,56^. In *crbRNAi* cells we found that Myo is missing from the gaps depleted in E-cad and F-actin (Fig. 7g), while strongly accumulating in rings around these gaps (Fig. 7g–l, arrowheads). In *yurtRNAi* cells, Myo was diffuse in the cytoplasm, being completely lost from the SAR (Fig. 7m–o) and correlating with an increase in the average vertex-vertex distance. Noticeably, a decrease in Myo has been previously associated to a loss of contractility and to an increase in junction length in pupal wing cells^56^.

Taken together, our results show that Crb is required for the proper localization of Yurt at the SAR at the end of hexagonal packing. The similar defects following *crb* or *yurt* knockdown on the distribution of both E-cad and actomyosin cytoskeleton at the AJs further suggests a common function in the stabilization of junctions during pupal wing morphogenesis.

## Discussion

Our study aimed at unveiling the function of the Crumbs complex in epithelial morphogenesis. Although Crb was discovered several decades ago in *Drosophila* ^7^, the severe apico-basal polarity defects associated to *crb* inactivation in embryos have hampered the full exploration of its function during epithelia development. Our results indicate that Crb also acts during pupal wing morphogenesis, where the absence of *crb* function does not impair AP/BL polarity and does not lead to the dramatic tissue alterations often seen in other tissues. The pupal wing thus represents an attractive model system, well suited to dissect additional functions of the Crb complex during epithelial morphogenesis, independently of its role in polarity.

The redistribution of Crb at the SAR at the end of hexagonal packing, as well as the defects in cells orientation observed in *crb* mutants suggest that Crb is required to stabilize the actin cytoskeleton and E-cadherin at the adherens junctions at the end of tissue rearrangement. Alterations in F-actin and Myo distribution in *crb* mutant cells strikingly mimic those observed in embryos mutant for the actin-binding protein Canoe/Afadin, which links the actomyosin network to AJs^54^. Canoe loss diminishes this coupling leading to reduced cell shape anisometry and defects in germ band elongation. As for *crb*, *canoe* mutant cells still retain some ability to change their shape and germ band elongation is delayed and not completely impaired. The defects observed in *crb* mutant cells support the hypothesis that Crb is a crucial regulator of the interconnection between the actomyosin cytoskeleton and AJs.

The fragmentation of AJs upon Crb depletion has been already described, for example in embryo^2,17^ or during follicular morphogenesis^16^. However, in these two systems the function of Crb has been related to the role of Moe in the regulation of the actomyosin cytoskeleton, while the role of Yurt has never been addressed or has been excluded. Our data support that in pupal wing cells the role of Crb in the stability of the AJs is likely established via Yurt. We show that Crb modulates Yurt localization at the SAR at the end of hexagonal packing and *yurt* mutant cells phenocopy *crb* mutant cortical defects. Nonetheless, previous studies in cultured cells have established that Yurt participates in epithelial polarity and organization of apical membranes by negatively regulating the activity of the Crb complex^15,57^. On the contrary, we show that, whereas Crb modulates Yurt distribution at the SAR at the end of hexagonal packing of wing cells, Yurt depletion does not impact Crb association to the SAR, with the exception of the E-cad- and F-actin-devoid gaps. Yurt and Crb similarly act on actomyosin and E-cad organization at the cell-cell junctions suggesting that the coordinated function of these two proteins is regulated by different mechanisms in different tissues. On the other hand, *moe* depletion does not specifically modify Crb distribution at the SAR, a finding coherent with the evidence that Moe is not implicated in stability of AJs in this tissue, as opposed to other models^16^.

Studies based on *in vivo* mechanical measurements or mathematical/physical modeling have proposed that epithelial cell packing results from a balance between intrinsic cell tension and extrinsic tissue-wide forces to establish a correct and robust order in the tissue^44,46,58,59^. Hence, the tension generated by the actomyosin cortex and the pressure transmitted through adherens junctions are the two main self-organizing forces driving tissue morphogenesis. Tension shortens cell-cell contacts and pressure of individual cells counteracts tension to maintain cell size^44,48,60,61^. Our data indicate that Crb recruits at SAR Moe and Yurt, which show opposite effects on pupal wing morphogenesis. While Moe promotes cell expansion, Yurt controls cell constriction and the stability of the AJs and of the actomyosin network. In *crb* mutant cells, the absence of variation in the cell perimeter might be explained by the simultaneous loss of positive and negative regulators. Therefore, Crb acts as a coordinator of the two self-organizing mechanisms implicated in morphogenesis. Additionally, the dynamic redistribution of Crb at the SAR at the end of hexagonal packing, together with the disruption of cell orientation in *crb* mutants, is consistent with the hypothesis that Crb is required to stabilize cell shape and pattern in order to properly progress throughout tissue development.

In conclusion, these functional analyses during pupal wing morphogenesis allowed us unraveling Crb-dependent mechanisms that are integrated to produce shape changes during development independently of epithelial polarity. Furthermore, our results show that the interplay between Crb and FERM proteins is tissue-regulated and that their epistatic interactions differ in a spatio-temporal manner.

## Methods

### *Drosophila* stocks and crosses

Control and driver strains (Bloomington Drosophila Stock Center, Indiana University); Transgenic lines used were *UAS-crb RNAi* (line 39177), *UAS-Yurt RNAi* (VDRC 107016 and 26674), (Vienna Drosophila RNAi Center, Vienna, Austria). These transgenic lines were crossed to *ptc-GAL4* at 25 °C.

Mutant strains were c*rb* ^*11A22*^ ^7^; *Sdt* ^*k85*^ ^62^; *DPatj* ^*53*,^ ^63^, *Moe* ^*PL106*^ ^51^. Mutant clones were generated using the FLP/FRT technique^64^. Crosses were grown at 25 °C and clones were recovered from pupae of the following genotypes:

*hs* > *flp*;; *FRT82B*, *crumbs* ^*11A22*^ */FRT82B, ubi* > *GFP*;

*Δmoe FRT19A/ubi* > *mRFP, hs* > *flp FRT19A*

*Sdt* ^*k85*^ *FRT19A/ubi* > *mRFP; hs* > *flp FRT19A*

*hs* > *flp; DPatj* ^*53*^ *FRT2A/ubi* > *GFP, FRT2A*

Mutant clones were generated by heat-shocking L2 larvae for 1 h at 37 °C. Pupae were dissected at 16–18, 28–30 or 32 h APF. Staging of pupal wing development at 25 °C were performed as described^39,65^.

*CG12075* coding for *Moesin* extends from 8767045 to 8792365 bp on the X chromosome. A null allele was generated by targeted deletion of the *Moesin* coding region using site specific recombination between two Piggy Bac elements [e02421] and [e04400] (Exelixis) as described in^66^. The resulting deficiencies, carrying the recombinant (hybrid element), were characterized molecularly by PCR using transposon or genomic specific primers according to^67^. The recombinant hybrid element was subsequently eliminated by precise excision and the resulting null allele for *Moesin*, selected on the basis of white eyes (loss of *mW*+), was characterized molecularly by PCR and sequencing.

### Immunofluorescence and antibodies

The head and the bottom of the pupae was dissected in PBS and quickly transferred in PFA 4% at room temperature for one hour. Dissected pupae were transferred into PBS-TN (PBS-0.3% Triton-20% NGS), the pupal case was removed and finally the wing was extracted. Washes were done in PBS-TN. Primary antibodies were incubated in PBS-TN, overnight at 4 °C. Secondary antibodies were incubated in PBS-TN for 1 hour at room temperature.

For Crb localization at SAR antibody staining in pupal wings were performed as previously described^39,68^ by using PBS 0.01% Triton X-100. Primary antibodies were: mouse anti-Fmi [1:20], mouse 4F3 anti-Dlg and rat anti-DE-Cad2 [1:100] from Developmental Studies Hybridoma Bank (DSHB, University of Iowa, USA); rat anti-Crumbs2-8^7^, rabbit anti-DPatj^69^, rat anti-Sdt^62^, rabbit anti-Moesin^70^, rat anti-Yurt^15^; anti-Myosin II^71^. Secondary antibodies and Rhodamine-conjugated phalloidin were from Molecular Probes and Jackson ImmunoResearch Laboratories. Confocal images were acquired at 40x, 63x and 100x magnification on a LSM 510 Zeiss Confocal Microscope. Confocal sections were spaced 0.5 μm apart.

### Quantifications

All quantifications were done on single optical sections corresponding to the AJ plane. For image analysis (cell perimeter, vertex-vertex distance, vertex number per cells, Crb mean intensity at SAR) we used the software “packing analyzer v2.0”^45^. Measurements of vertex-vertex length over time were done manually using ImageJ, and were normalized by its average vertex-vertex length over time.

For quantification of the orientation angle of hexagonal cells, cells were segmented and then analyzed with a custom written Matlab code based on the regionsprops function. Briefly, based on the segmented images, an ellipse was fitted on each cell (see below) and different parameters were extracted, such as the length of the Major and Minor axis allowing computing both the eccentricity and the orientation of the ellipse.

Cell eccentricity was computed using this equation (1):

$${\rm{Cell}}\,{\rm{eccentricity}}=2\times \surd ({(Lengt{h}_{MajorAxis}/2)}^{2}-{(Lengt{h}_{MinorAxis}/2)}^{2}/Lengt{h}_{MajorAxis}$$

If cells are totally round with a Length_MajorAxis_ = Length_Minor Axis_, eccentricity value is 0. In this study we used 0.5 as threshold for an elongated (>0.5, Cell 1 blue, below) and anisotropic cell (<0.5, Cell 2 green, Table 1, below).

**Table 1:** The datasets analysed in the current study are available from the corresponding author on request.

	Max Length	Min Length	Eccentricity	Orientation
Cell 1 (blue)	77.4981592	48.6524703	0.77838379	8.46648541
Cell 2 (green)	59.2989365	58.4526612	0.16834195	30.3486546

The ellipse orientation (in degrees ranging from 0° to 90°) is defined as the angle between the vein 3, which define the P/D axis, and the major axis of the ellipse, allowing to discriminate between a random oriented cell (angle >25°) and cell oriented in the plane of the wing elongation (angle <25°). We considered for quantification: for *wt* and *crbRNAi* 20 cells per wing, n_wings_ = 9 with total n_cells_ analyzed = 180; and for c*rb* ^*11A22*^ and its *wt* twin clone 20 cells per wing, n_wings_ = 3 with total n_cells_ analyzed = 60.

## Electronic supplementary material

Supplementary information

Movie 1

Movie 2
