# Supplementary material for: Crumbs, Moesin and Yurt regulate junctional stability and dynamics for a proper morphogenesis of the Drosophila pupal wing epithelium
Source: Sci Rep. 2017 Dec 1;7:16778. doi: 10.1038/s41598-017-15272-1 (PMC5711895; doi:10.1038/s41598-017-15272-1)
Supplement: Supplementary file 1 — Supplementary information [file 41598_2017_15272_MOESM1_ESM.pdf]

Crumbs, Moesin and Yurt regulate junctional stability and dynamics for a proper morphogenesis of the *Drosophila* pupal wing epithelium

Pauline Salis[1§], Francois Payre[2], Philippe Valenti[2], Elsa Bazellieres[1], André Le Bivic[1*&] and Giovanna Mottola[1,#, &]

[1]Aix-Marseille Université, CNRS UMR 7288, Developmental Biology Institute of Marseille Luminy (IBDM), Marseille, France.
[2]Centre de Biologie du Développement, CNRS UMR5547, Université Paul Sabatier, Toulouse, France
[#]Present addresses : Aix-Marseille University, UMR MD2 and IRBA, Marseille, France; Laboratory of Biochemistry, La Timone University Hospital, Marseille, France
[§]Present address: UMR CNRS 7232 OOB, Université Pierre et Marie Curie, Banyuls-sur-Mer, France

[&]co-last authors
*Correspondence: andre.le-bivic@univ-amu.fr

Supplementary Figures Legends

Figure S1. Sdt, but not DPatj, determines Crb localization to the SAR and participates in prehair development.

(a-f) sdt^{K85} clones in pupal wings at 32-34 h APF, indicated by the absence of GFP (blue), stained for Crb (green) and F-actin (red). (g-l) $DPatj^{53}$ clones in pupal wings at 25°C at 32-34 h APF, indicated by the absence of DPatj (blue), stained for Crb (green) and F-actin (red). All images are maximal projections of 2 up to 6 optical sections (every 0.2 μm). Distal is right, proximal left. Scale bar: 10 μm.

Figure S2. Crb is not required for apical/basal and planar polarities of the pupal wing epithelium.

(a-l) Dlg (green), F-actin (red) and E-cad (blue) distribution in *wt* (a-c and g-i) and *crb-RNAi* (d-f and j-l) pupal wings at 25°C at 30 h APF; (g-l) orthogonal section of pupal wings. (m-r) Fmi (green) and F-actin (red) distribution in *wt* (M-O) and *crb-RNAi* (P-R) pupal cells at 30 h APF. Red dots in panels m and p show the Fmi zig-zag pattern oriented orthogonally to the P/D axis. On the right of panels a-f and m-r drawn orthogonal views of a wing epithelial cell where the focal plane positions of the confocal image projections in the left panels are indicated (black line). All images are maximal projections of 2 up to 6 optical sections (every 0.2 μm). Distal is right, proximal left. Scale bar: 10 μm.

Figure S3. Crb is required for the stability of cell perimeter fluctuations

(a) Quantification of cell perimeter length (μm) in *wt*, *crbRNAi* and crb^{11A22} cells at 28-30 h APF. Bars indicate mean values ± SEM and statistical significance was analyzed by Student's t-test [*crbRNAi* (11.78 μm ± 0.10) versus *wt* cells (12.25 μm ± 0.08), $n_{cells} = 200$, $P < 0.05$; crb^{11A22} inside the clone (11.64 μm ± 0.24) versus *wt* cells (11.40 μm ± 0.16) $n_{cells} = 80$, $P > 0.05$ (n.s.); crb^{11A22} at the border of the clone (10.62 μm ± 0.27) versus *wt* cells (10.14 μm ± 0.23; $n_{cells} = 70$, $P > 0.05$ (n.s.)]. (b) Quantification of the vertex-vertex distance in *wt*, *crbRNAi* and crb^{11A22} cells at 28-30 h APF. Bars indicate mean values ± SEM and statistical significance was analyzed by Student's t-test [*crbRNAi* (1.95 μm ± 0,04) versus *wt* (1.98 μm ± 0,04) junctions $P > 0.05$ (n.s.); crb^{11A22} inside the clone (2.03 μm ± 0,08) versus *wt* cells (2.30 μm ± 0,06) $P < 0.05$; crb^{11A22} border (2.28 μm ± 0,08) versus *wt* cells (2.25 μm ± 0.08) $n_{cells}=200$, $P > 0.05$).

(c) Example of variation of cell perimeter fluctuations by *in vivo* imaging of E-cad-GFP in *wt* and *crbRNAi* contexts. Cell perimeter length is color-coded (heat map from red to yellow) based on the percentage of cell perimeter length decrease calculated with respect to the cell perimeter length captured during imaging. (d) Graph of evolution of cell perimeter variation amplitude in *wt* (red) and *crbRNAi* (black) tissues. Each cell perimeter is normalized by its average length over time and expressed in arbitrary units (A.U.).

Figure S4. Characterization of *moe* null mutants.

(a-f) Pupal wings containing moe^{PL106} clones at 28-30 h (a-c) and 32 h (d-f) APF. Moe depletion is revealed by the absence of Moe staining (blue). F-actin (red) staining does not reveal defects in apical F-actin redistribution or pre-hair organization**.** All images are maximal projections of 2 up to 6 optical sections (every 0.2 μm). Distal is right, proximal left. Scale bar: 10 μm. (g) Genomic organization of the *moesin* gene, flanking genes (CG12075 and CG1885) and transposable elements ($PBac^{e02421}$ and $PBacMoe^{e04400}$) used to generate the 25.32 kb deletion (*Df(X)Moesin*, called *Δmoe*) (*72*).

Figure S5. Characterization of Sdt, E-cad and Fmi staining in *moe* null mutants.

(a-f) Pupal wings containing *Δmoe* clones at 28-30 h and stained with Sdt (green) (a-b), E-cad (green) (c-d) and Fmi (green) (e-f). *Δmoe* clones are indicated by the absence of GFP (blue). (g) Quantification of Fmi staining at the cortex in *wt* and *Δmoe* clones (in the center of the clone and along the border of the clone) in pupal wings at 28-30 h APF. Fmi intensity per length unit (pixel intensity average) at the apico-lateral cortex was calculated and expressed in A.U. Bars indicate mean values of intensity ± SEM and statistical significance was analyzed by Student's t-test (*Δmoe* = $6.29 \pm 0.08 \times 10^6$ A.U., versus *wt* = $6.06 \pm 0.08 \times 10^6$ A.U., $n_{wings}=5$, $n_{cells}=100$; $P > 0.05$).

Movie 1. Time-lapse movie of wt and *crbRNAi* pupal wing expressing E-cad::GFP at 28-30 h APF. Note the appearance and disappearance of E-cad::GFP gaps and the higher variability of cell perimeter length in *crbRNAi*, compared to *wt*.

Movie 2. Time-lapse movie of *Δmoe* pupal wing expressing E-cad::GFP at 28-30 h APF. Note the higher constriction of cell perimeter length and the absence of E-cad::GFP gaps.

Figure S1

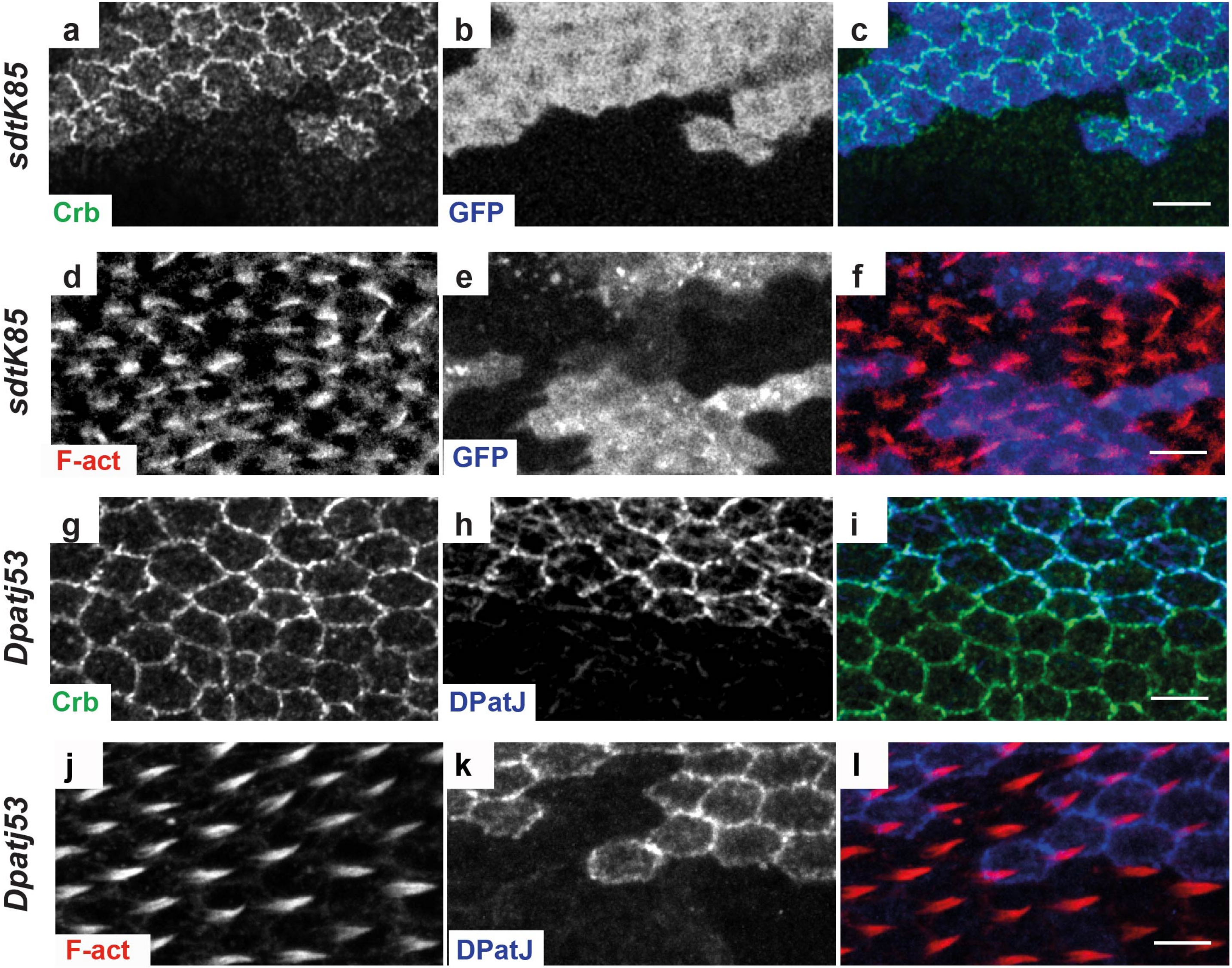

a
b
c
sdtK85
Crb
GFP
d
e
f
sdtK85
F-act
GFP
g
h
i
Dpatj53
Crb
DPatJ
j
k
l
Dpatj53
F-act
DPatJ

Figure S2

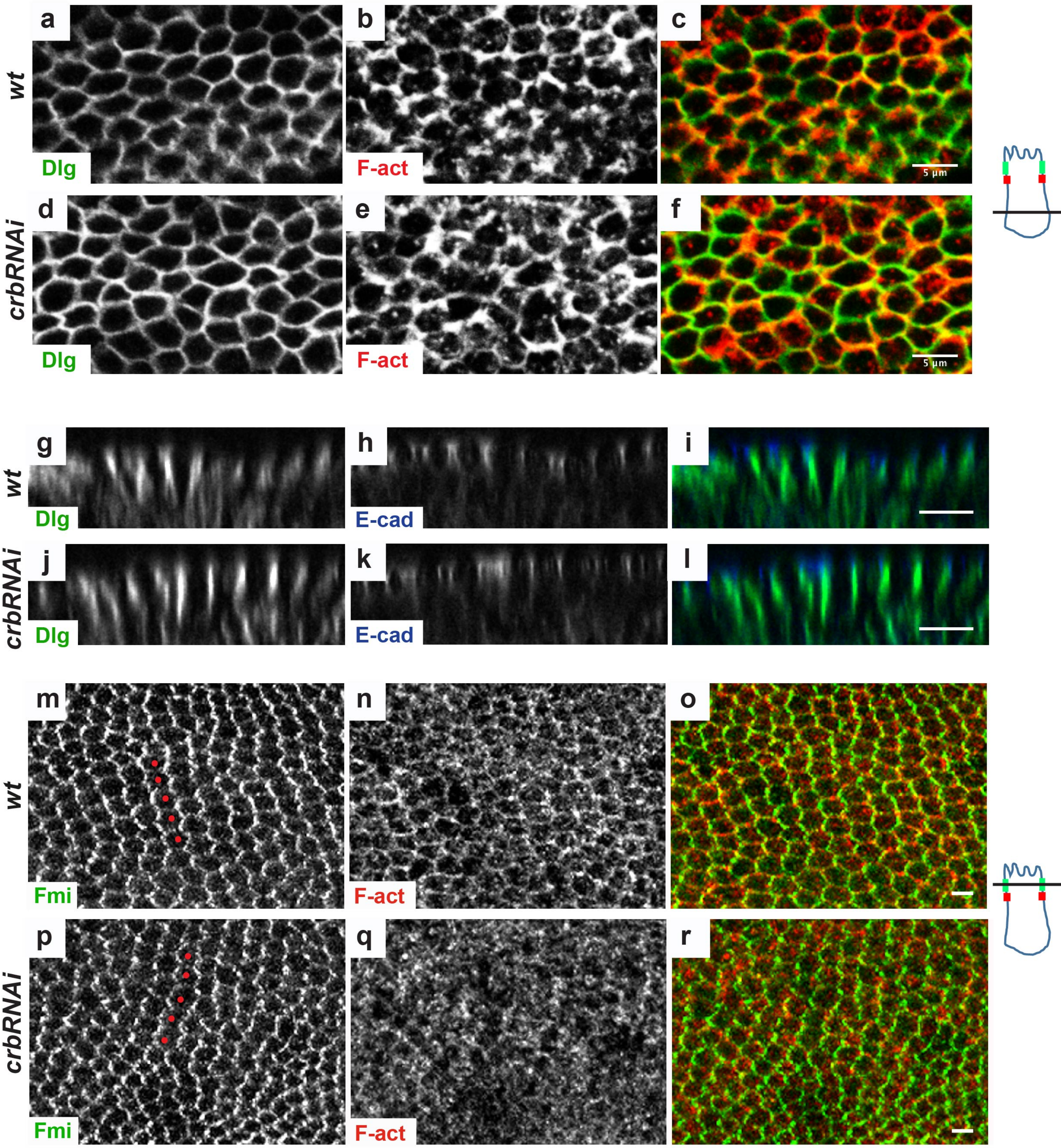

a
b
c
wt
Dlg
F-act
5 µm
d
e
f
crbRNAi
Dlg
F-act
5 µm
g
h
i
wt
Dlg
E-cad
j
k
l
crbRNAi
Dlg
E-cad
m
n
o
wt
Fmi
F-act
p
q
r
crbRNAi
Fmi
F-act

Figure S3

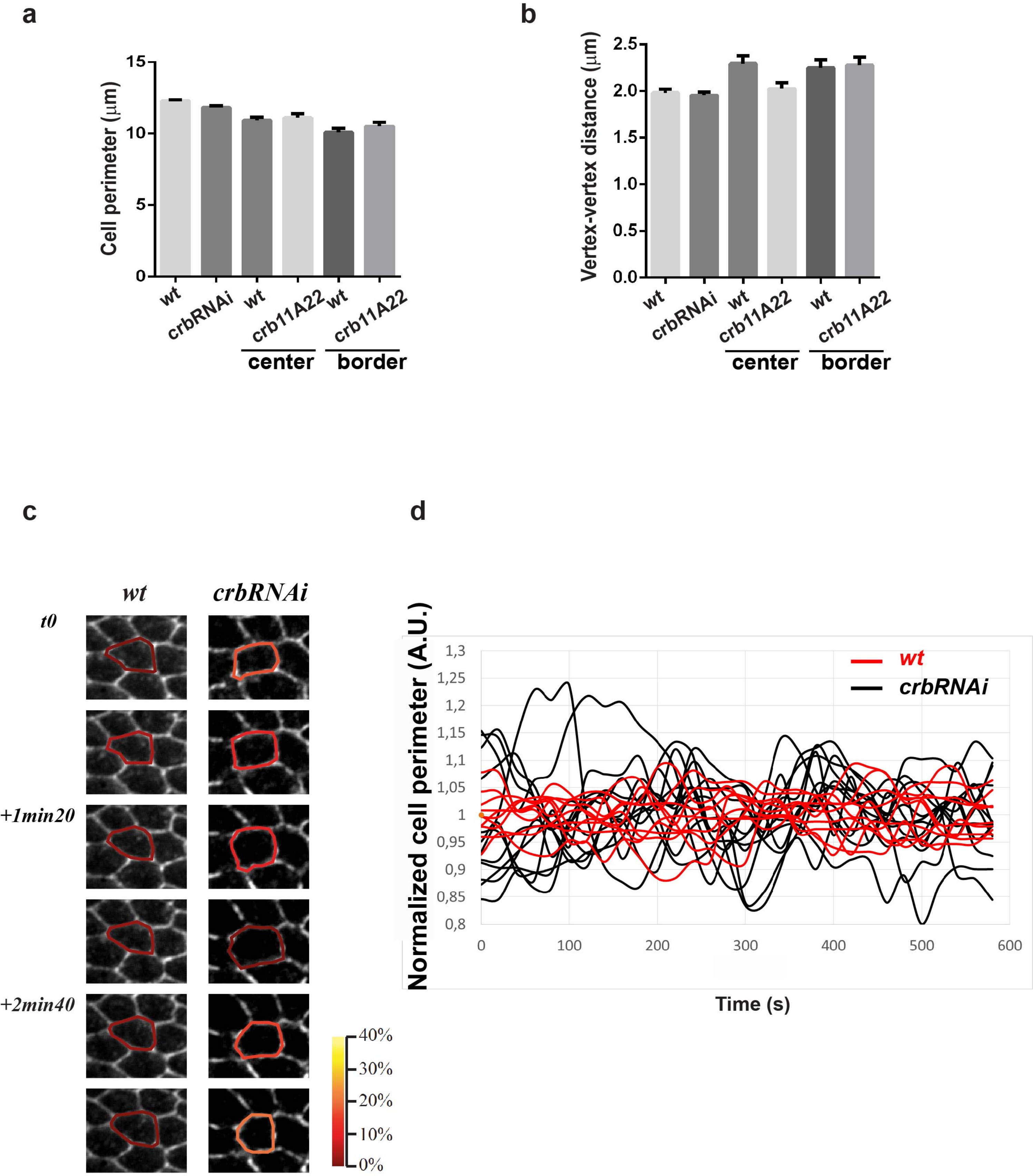
a
Cell perimeter (µm)
15
10
5
0
wt
crbRNAi
wt
crb11A22
wt
crb11A22
center
border
b
Vertex-vertex distance (µm)
2.5
2.0
1.5
1.0
0.5
0.0
wt
crbRNAi
wt
crb11A22
wt
crb11A22
center
border
c
wt
crbRNAi
t0
+1min20
+2min40
40%
30%
20%
10%
0%
d
Normalized cell perimeter (A.U.)
1,3
1,25
1,2
1,15
1,1
1,05
1
0,95
0,9
0,85
0,8
0
100
200
300
400
500
600
Time (s)
wt
crbRNAi

Figure S4

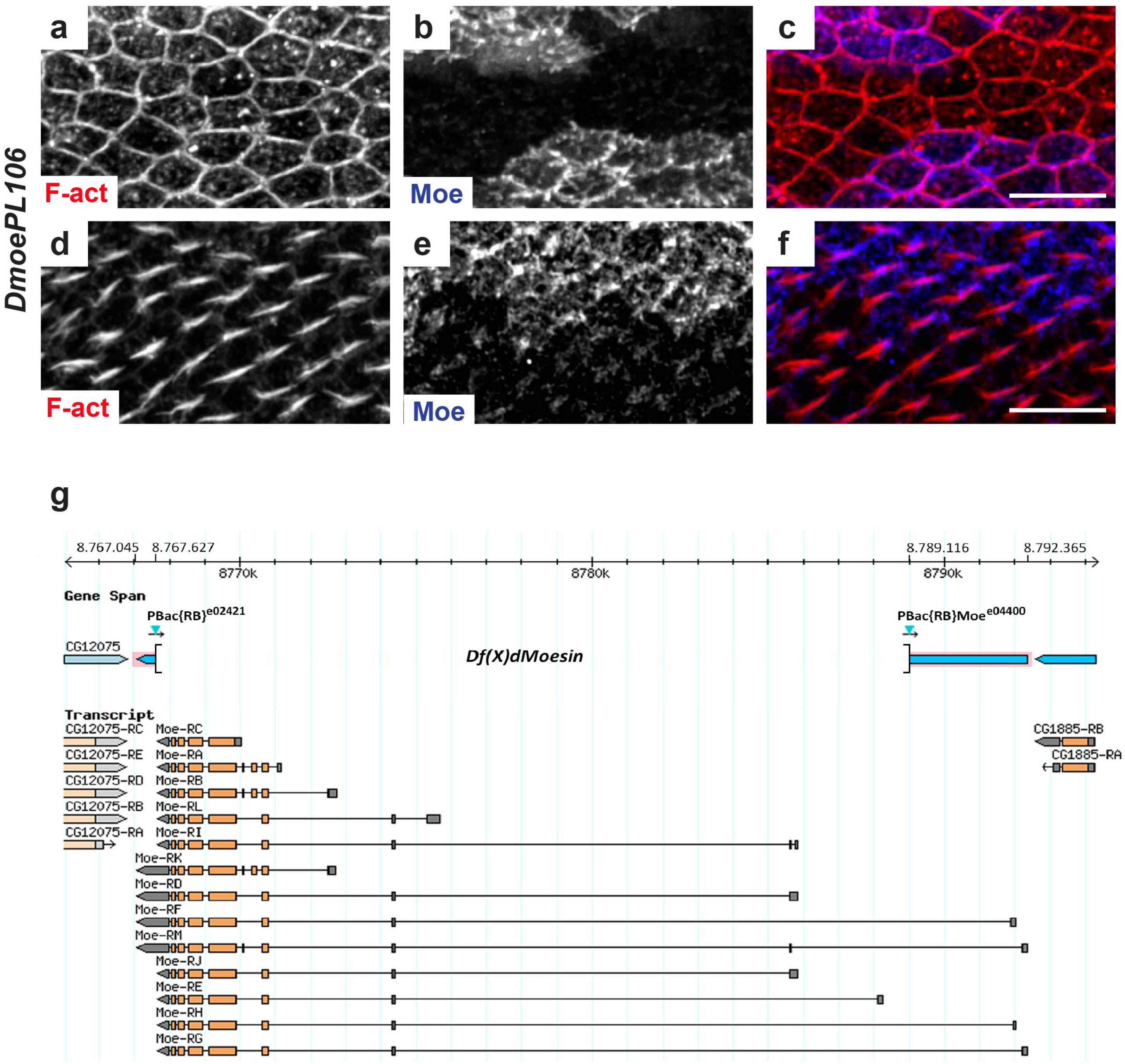

a
b
c
F-act
Moe
DmoePL106
d
e
f
F-act
Moe
g
8.767.045
8.767.627
8770k
8780k
8.789.116
8790k
8.792.365
Gene Span
PBac{RB}e02421
PBac{RB}Moee04400
CG12075
Df(X)dMoesin
Transcript
CG12075-RC
CG12075-RE
CG12075-RD
CG12075-RB
CG12075-RA
Moe-RC
Moe-RA
Moe-RB
Moe-RL
Moe-RI
Moe-RK
Moe-RD
Moe-RF
Moe-RM
Moe-RJ
Moe-RE
Moe-RH
Moe-RG
CG1885-RB
CG1885-RA

Figure S5

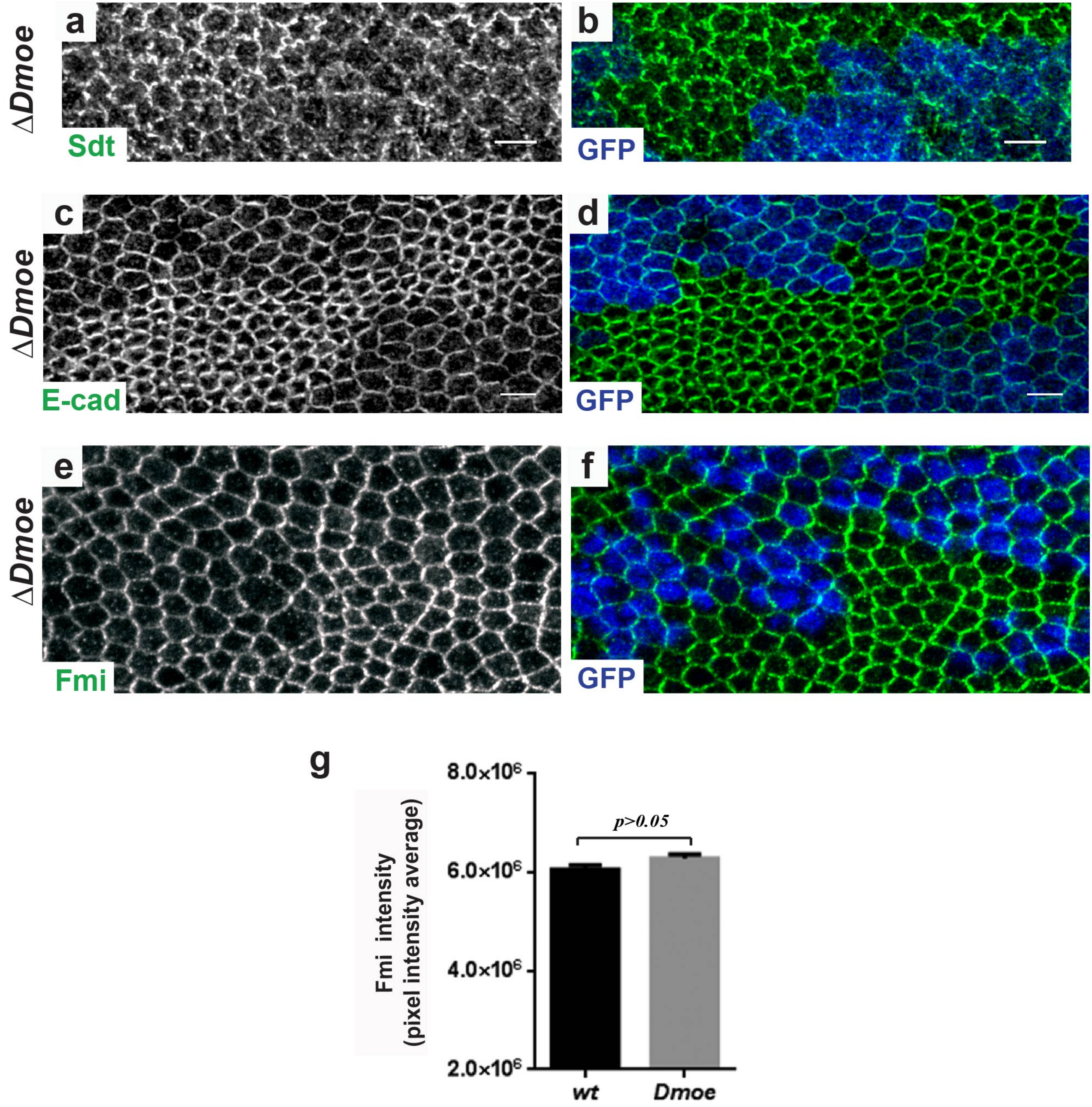

ΔDmoe
a
Sdt
b
GFP
ΔDmoe
c
E-cad
d
GFP
ΔDmoe
e
Fmi
f
GFP
g
Fmi intensity
(pixel intensity average)
8.0×10^6
6.0×10^6
4.0×10^6
2.0×10^6
p>0.05
wt
Dmoe
